# National Smoking Cessation Services (NSCS) enrollment and their effect on long-term tobacco cessation in Korea: Results from a 1-year prospective follow-up of NSCS participants

**DOI:** 10.18332/tid/178499

**Published:** 2024-02-08

**Authors:** Jinju Park, Min Kyung Lim, Yunhee Kim, Yu-Jin Paek, Sung-il Cho

**Affiliations:** 1 Central Division of Cardio-cerebrovascular Disease Management, Seoul National University Hospital, Seoul, Republic of Korea; 2 Department of Social and Preventive Medicine, Inha University College of Medicine, Incheon, Republic of Korea; 3 Department of Nursing, Pukyong National University, Busan, Republic of Korea; 4 Department of Family Medicine, Hallym University Sacred Heart Hospital, Anyang, Republic of Korea; 5 Department of Public Health Sciences, Seoul National University Graduate School of Public Health, Seoul, Republic of Korea

**Keywords:** smoking cessation, long-term effect, national cessation service

## Abstract

**INTRODUCTION:**

The study aimed to identify predictors associated with long-term tobacco cessation at 12-month follow-up among users of the National Smoking Cessation Services (NSCS) in Korea.

**METHODS:**

To measure the long-term effect of NSCS delivery, the target sample size of 5167 adult smokers registered in the NSCS in 2018 was enrolled with proportional random sampling. A multiple logistic regression analysis (crude, adjusted) was performed to identify the changes in smoking status at the 12-month follow-up after the last NSCS enrollment and the potential factors associated with changes in smoking status.

**RESULTS:**

The response rate to reach the number of subjects targeted was 22.4%. A total of 41.2% of the tobacco users enrolled had successfully quit at baseline, and the 7-day point prevalence of tobacco cessation at the follow-up at 12 months, via a telephone survey, was 34.4%. Factors positively associated with cessation at the 12-month follow-up were longer experience with tobacco abstinence and additional quitting attempts with or without NSCS enrollment, although every additional quit attempt with NSCS use had a better outcome. In addition, having a successful quit outcome with NSCS use at the baseline and having more satisfaction with the service of professional counseling or incentives than others provided by NSCS, increased cessation at follow-up after adjustment of other factors considered.

**CONCLUSIONS:**

In addition to multiple quitting attempts, longer experience with tobacco abstinence, and additional enrollment in NSCS, the service experience, and satisfaction with the content that NSCS offered, might improve the lasting success of abstinence. These results might be considered to improve the contents and protocols of the NSCS for better outcomes.

## INTRODUCTION

The World Health Organization (WHO) Framework Convention on Tobacco Control (FCTC) suggested ‘offering cessation’ as a key tobacco control measure, and many countries have since developed and implemented smoking cessation services^1,2^. In the Republic of Korea, the National Smoking Cessation Services (NSCS) began with the launch of the public health center-based smoking cessation clinics (PHCs) in 2005. The establishment of the PHCs was followed by the establishment of a telephone-based smoking cessation service, Quitline (QL), which was launched in 2006. With the increased funding received from a tobacco tax levied starting in 2015, which doubled the consumer price of tobacco, various channels and comprehensive models have been developed and applied for increases in coverage and service reach.

The PHCs are a government-supported nationwide smoking cessation service run by 255 public health centers across the country. This service is easy to access at the community level and offers affordable and comprehensive services providing nicotine replacement therapy (NRT) and/or prescription drugs, and professional counseling. In addition, it is compulsory to measure carbon monoxide (CO) or urinary cotinine levels when smokers are undergoing face-to-face counseling.

QL is a telephone-based multisession counseling service that provides systematic, comprehensive, target-specific behavioral counseling for men, women, and adolescent smokers with a 1-year service protocol. A quitting guide booklet and a quit pack are mailed to the smoker, and additional services are provided by e-mail and text message.

Mobile smoking cessation clinics (MCs) were developed for vulnerable populations that may be more likely to face particular challenges while trying to quit; these populations include out-of-school youths, university students, women, disabled persons, and employees exposed to heavy smoking in small businesses. MC services include nine intensive behavior counseling sessions and NRTs over six months, and participants’ CO levels are measured to help maintain smoking cessation.

Individual-tailored intensive smoking cessation services in hospitals (ITIS) were developed with a benchmark of an 8-day hospitalization program in the Nicotine Dependence Center of the Mayo Clinic and offer individual-tailored intensive treatment services, such as medical assessments and daily medical follow-up, educational sessions to help participants quit and maintain their quitting status, addiction treatment with drugs, and professional counseling for behavioral changes over a 5-day period. General smoking cessation education and counseling services (GECS) include 2-day individual and group education programs and/or counseling sessions by quit coaches.

Smoking cessation treatment services in hospitals/clinics (TSH) are available in hospitals and clinics that have joined the smoking cessation programs operated by the National Health Insurance Service (NHIS) and receive funding support from the government. For 12 weeks, patients receive six counseling sessions and prescription drugs and/or NRT for 28 days per month (up to 84 days), which are offered by health professionals. This service can be used up to three times per year when relapses occur.

Although each cessation service offers different contents, channels, and schedules, the overall impact on the reduction of smoking prevalence should be monitored to determine the success of long-term smoking cessation and the factors associated with this success among the participants of each cessation service. The effects of telephone-based cessation services in North America and ‘Stop Smoking Cessation Services’ in the United Kingdom have been evaluated in terms of improving short- and long-term quitting rates and decreasing tobacco prevalence^3-5^. Approximately 10% of annual successful quitters relapse after one year, as reported in a meta-analysis which was conducted to evaluate lifetime smoking cessation^6^. Previous studies have shown that continued abstinence at the 12-month follow-up is a good predictor for long-term abstinence^7^.

Though some previous studies have discussed the long-term success rates of these services and found several predictors of long-term success in smoking cessation, the findings were either based on the outcome measures determined at the end of each service use or were not derived from real-world settings. Moreover, there have been no studies of the various types of cessation services applied in a single country, such as Korea, at the national level, emphasizing the need to measure the overall effects of all types of cessation services.

With the availability of nationally representative data on NSCS-registered participants and their follow-up through 12 months, the current study aimed to analyze the effects of NSCS on quitting outcomes just after completing the NSCS program and on long-term tobacco cessation at the follow-up at 12 months. Furthermore, this study was conducted to identify the predictors associated with changes in smoking status after NSCS enrollment and long-term tobacco cessation in Korea.

## METHODS

### Study populations

National data on smokers registered in the NSCS were obtained from the National Center for Cessation Support, Korea Health Promotion Institute. In brief, proportional random sampling was used to stratify participants to ensure a diverse mix of age, sex, and area of residence across each type of cessation service. To avoid possible selection bias, weight defined by proportions of enrollment of each type of cessation service was used to stratify and adjust the representation of the final participants using data from proportions of enrollment in the NSCS in 2018. Ultimately, 5167 adult former smokers who had registered for and participated in the NSCS in 2018 were included in the final analysis (Supplementary file Table 1).

### Study procedures

The survey was performed between 13 and 27 March 2020, by highly trained interviewers with experience in telephone surveys and was managed by the Department of Health Administration office of the NHIS and by the survey company Korea Management Association Consultants Inc., for participants in the TSH and participants in other NSCS, respectively. Telephone-based surveys were conducted with five times the target populations to meet the target sample size of 5167 (N=15110 for the survey company KMAC and N=10725 for the NHIS). The number of smokers considered at each baseline outcome were 2129 for the tobacco abstinence group (1101 for PHC, 22 for MC, 14 for ITIS, 5 for GECS, 6 for QL, and 981 for TSH) and 3038 for the tobacco non-abstinence group (1753 for PHC, 95 for MC, 9 for ITIS, 6 for GECS, 11 for QL, and 1164 for TSH). The survey was conducted by determining the 7-day point prevalence of abstinence as a first question, as different forms of questions were employed, to account for the transitions. We obtained verbal informed consent from all participants to conduct the interviews. Institutional Review Board (IRB) approval (National Cancer Center Korea IRB NCC2020-0013) was obtained for this study.

### Measures

Gender, age, area of residence, type of NSCS in which the smoker participated, and quitting outcome upon completion of the NSCS program were identified from the data from the NSCS database at the baseline of the study to confirm the target study participants and were included as analytical variables.

Information on the status of tobacco use at the follow-up at 12 months, additional quitting attempts during the follow-up, number of additional quitting attempts during the follow-up (only for participants who had ever attempted to quit previously), length of longest tobacco abstinence ever maintained during the follow-up, most satisfactory service contents from the NSCS, most helpful contents from the NSCS for current tobacco cessation attempt, and type of tobacco product used before attempting to quit through the NSCS were also measured through the telephone interview as self-reported at the follow-up at 12 months. Furthermore, four types of questionnaires were developed based on the quitting outcomes at baseline and at the 12-month follow-up to consider the factors associated with transitions in tobacco use between the baseline at the end of the NSCS, when participants were enrolled and 12 months after the participants were enrolled.

The 7-day point prevalence of cessation at the follow-up at 12 months was assessed using the question: ‘Did you smoke during the past seven days?’. The quitting outcomes upon completion of the NSCS program at baseline were categorized into two groups: ‘abstinence’ and ‘non-abstinence.’ The status of tobacco use at the end of the 12-month follow-up was differentiated into ‘participant in tobacco cessation’ and ‘participant in tobacco use’.

### Statistical analysis

The baseline tobacco abstinence rate and the 7-day point prevalence of cessation at the 12-month follow-up were calculated and provided. Baseline characteristics were compared across the participants by the status of tobacco abstinence at the end of the NSCS program using chi-squared tests for categorical variables. Two-sided p<0.05 was considered statistically significant. Furthermore, multiple logistic regression analyses were performed to produce crude odds ratios (ORs), adjusted odds ratios (AORs), and 95% confidence intervals (CIs). ORs and AORs were produced to identify the factors associated with tobacco use at the 12-month follow-up among the abstinence group at baseline (Table 3) and the factors associated with tobacco cessation at the 12-month follow-up among the non-abstinence group at baseline (Table 4). Age and gender were covariates included in the model due to their associations with tobacco use in previous literature^8^. All the analyses were performed using SAS software version 9.4 (SAS Institute Inc.).

## RESULTS

**Figure 1:**
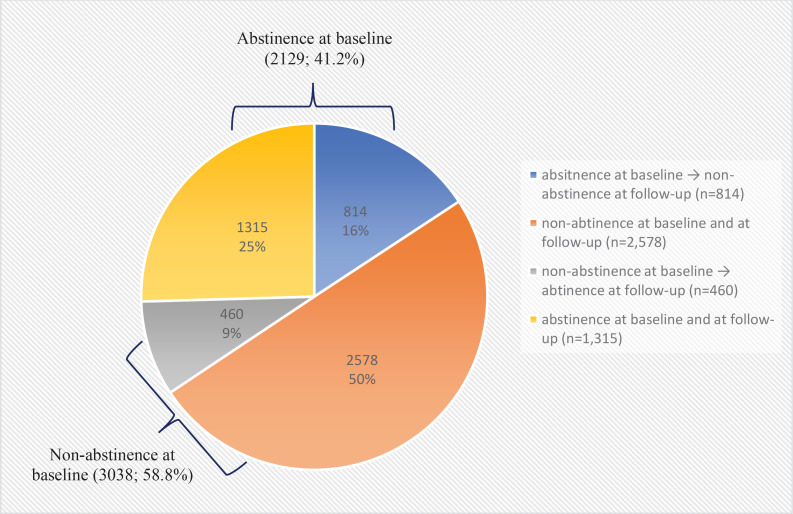
Changes in smoking cessation status at baseline and at follow-up at 12 months, of adult smokers registered in the NSCS in 2018, results from a prospective follow-up, 2018–2020 (N=5167)

Telephone-based surveys were conducted with five times the target populations and resulted in a survey response rate of 22.4%. Among the total enrolled population (N=5167), 41.2% of tobacco users enrolled had successfully quit at baseline upon the completion of the NSCS program (Figure 1). The baseline characteristics of the participants according to the status of tobacco abstinence upon the completion of the NSCS program are presented in Table 1. A greater number of participants in the abstinence at baseline group reported to be male participants, aged >50 years, and reported prescription medications/NRTs as the most satisfactory contents of the NSCS compared to the non-abstinence group. Furthermore, a greater number of participants reported having longer past experiences with tobacco cessation among the abstinence group compared to the non-abstinence group.

**Table 1:** Baseline characteristics of adult smokers registered in the NSCS in 2018, by status of tobacco abstinence upon the completion of the NSCS program, results from a prospective follow-up, 2018–2020 (N=5167)

	*Total enrolled (N=5167) n (%)*	*Participants in non-abstinence^a^ (N=3038) n (%)*	*Participants in abstinence^b^ (N=2129) n (%)*	*p^c^*
**Gender**				
Male	4552 (88.1)	2653 (87.3)	1899 (89.2)	0.0446
Female	615 (11.9)	385 (12.7)	230 (10.8)	
**Age** (years)				
20–29	633 (12.3)	431 (14.2)	202 (9.5)	<0.0001
30–39	810 (15.7)	499 (16.4)	311 (14.6)	
40–49	1188 (23.0)	702 (23.1)	486 (22.8)	
≥50	2536 (49.1)	1406 (46.3)	1130 (53.1)	
Residence				
Metropolitan	2215 (42.9)	1314 (43.3)	901 (42.3)	0.5114
City and county	2952 (57.1)	1724 (56.7)	1228 (57.7)	
**Most satisfied services in NSCS ever enrolled**				
Information on how to quit	528 (10.4)	327 (11.1)	201 (9.5)	0.0007
Professional counseling/support	1370 (27.0)	817 (27.6)	553 (26.2)	
Prescription medications/NRTs	2411 (47.6)	1347 (45.5)	1064 (50.4)	
Gift/incentives/tool kits	616 (12.2)	366 (12.4)	250 (11.8)	
Other	144 (2.8)	102 (3.4)	42 (2.0)	
**Length of the longest tobacco abstinence ever kept during the NSCS program** (months)				
<1	1141 (22.1)	1020 (33.6)	121 (5.7)	<0.0001
1–3	877 (17.0)	688 (22.6)	189 (8.9)	
3–6	783 (15.2)	498 (16.4)	285 (13.4)	
>6	2366 (45.8)	832 (27.4)	1534 (72.1)	
**Type of tobacco product used before quit attempt through NSCS**				
Heated tobacco	239 (4.6)	155 (5.1)	84 (3.9)	0.1213
E-cigarettes	86 (1.7)	53 (1.7)	33 (1.6)	
Conventional cigarettes	4836 (93.6)	2828 (93.1)	2008 (94.3)	
Other tobacco product	6 (0.1)	2 (0.1)	4 (0.2)	

NRT: nicotine replacement therapy. NSCS: National Smoking Cessation Services.

a Participants who kept tobacco abstinence during and upon the completion of the NSCS program, which is the baseline when they were enrolled.

b Participants who failed to keep tobacco abstinence during and upon the completion of the NSCS program, which is the baseline when they were enrolled.

c Chi-squared test was performed.

As shown in Table 2, the 7-day point prevalence of tobacco cessation at the 12-month follow-up was 34.4% (N=1775). The abstinence at the baseline group showed a significant OR of 9.19 (95% CI: 8.04–10.50) for the 7-day point prevalence of tobacco cessation compared to the non-abstinence at baseline group. Those with longer previous experience with tobacco abstinence (≥6 months, OR=19.20; 95% CI: 15.08–24.46) and those with additional quitting attempts with (OR=3.41; 95% CI: 2.84–4.09) or without (OR=1.99; 95% CI: 1.66–2.38) NSCS enrollment at the time of relapse, were positively associated with the 7-day point prevalence of tobacco cessation at the follow-up at 12 months. Participants who answered that professional counseling/support or gifts/incentives/toolkits were the most satisfactory services provided by the NSCS were more likely to be in the tobacco cessation group at the follow-up at 12 months.

**Table 2:** Factors associated with a 7-day point prevalence of tobacco cessation at follow-up at 12 months, of adult smokers registered in the NSCS in 2018, results from a prospective follow-up, 2018–2020 (N=5167)

*Variables*	*Total enrolled (N=5167) n*	*Participants in tobacco cessation at follow-up at 12 months^a^ (N=1775) n (%)*	*OR (95% CI)*	*AOR (95% CI)*
**Gender**				
Male ®	4552	1548 (34.0)	1	1
Female	615	227 (36.9)	1.14 (0.95–1.35)	1.14 (0.96–1.36)
**Age** (years)				
20–29 ®	633	202 (31.9)	1	1
30–39	810	268 (33.1)	1.06 (0.85–1.32)	1.06 (0.85–1.32)
40–49	1188	390 (32.8)	1.04 (0.85–1.28)	1.05 (0.85–1.29)
≥50	2536	915 (36.1)	1.20 (1.00–1.45)	1.21 (1.01–1.46)
**Residence**				
Metropolitan ®	2215	769 (34.7)	1	1
City and county	2952	1006 (34.1)	0.97 (0.87–1.09)	0.97 (0.87–1.09)
**Baseline status of tobacco abstinence upon completion of the NSCS program**				
Fail to keep abstinence ®	3038	460 (15.1)	1	1
Succeed in keeping abstinence	2129	1315 (61.8)	9.05 (7.93–10.33)	9.19 (8.04–10.50)
**Most satisfied services in the NSCS program ever enrolled**				
Information on how to quit ®	528	170 (32.2)	1	1
Professional counseling/support	1370	520 (38.0)	1.29 (1.04–1.59)	1.29 (1.04–1.60)
Prescription medications/NRTs	2411	789 (32.7)	1.02 (0.84–1.25)	1.04 (0.85–1.28)
Gift/incentives/tool kits	616	234 (38.0)	1.29 (1.01–1.65)	1.36 (1.06–1.74)
Other	144	44 (30.6)	0.93 (0.62–1.38)	0.92 (0.62–1.37)
**Length of the longest tobacco abstinence ever kept during the NSCS program and 12-month follow-up** (months)				
<1 ®	1141	81 (7.1)	1	1
1–3	877	135 (15.4)	2.38 (1.78–3.19)	2.40 (1.79–3.21)
3–6	783	168 (21.5)	3.58 (2.69–4.75)	3.65 (2.75–4.85)
>6	2366	1391 (58.8)	18.67 (14.68–23.74)	19.20 (15.08–24.46)
**Experience additional quit attempts during the 12-month follow-up**				
Never even though relapsed ®	1882	264 (14.0)	1	1
Ever without NSCS enrollment	1360	330 (24.3)	1.96 (1.64–2.35)	1.99 (1.66–2.38)
Ever with NSCS enrollment	1056	382 (36.2)	3.47 (2.90–4.16)	3.41 (2.84–4.09)
Never as in prolonged abstinence	869	799 (91.9)		
**Type of tobacco product used before quit attempt through NSCS**				
Heated tobacco ®	239	92 (38.5)	1	1
E-cigarettes	86	30 (34.9)	0.86 (0.51–1.43)	0.84 (0.50–1.41)
Conventional cigarettes	4836	1650 (34.1)	0.83 (0.63–1.08)	0.78 (0.60–1.02)
Other tobacco product	6	3 (50.0)	1.60 (0.32–8.09)	1.53 (0.30–7.74)

a Participants in tobacco abstinence for the last 7 days at follow-up at 12 months upon the completion of last NSCS program enrolled. AOR: adjusted odds ratio; adjusted for age and gender. ® Reference categories. NRT: nicotine replacement therapy. NSCS: national smoking cessation services.

Factors associated with tobacco use at 12-month follow-up among the participants who succeeded in tobacco abstinence at baseline are presented in Table 3. Of the participants in the tobacco abstinence at baseline group, 38.2% had become tobacco users at the follow-up at 12 months. Those who answered that the most satisfactory contents of the NSCS were professional counseling/support showed a significant OR of 0.64 (95% CI: 0.45–0.90) compared to those who answered information on how to keep from relapsing back into tobacco use at the follow-up at 12 months. Furthermore, those who answered that the most satisfactory contents of the NSCS were medications/NRTs showed an inverse association (OR=1.45; 95% CI: 1.06–1.98). Participants with longer past experiences with tobacco abstinence were negatively associated with relapsing back into tobacco use at the 12-month follow-up (OR=0.08; 95% CI: 0.05–0.12 for six months or more versus less than one month). However, having additional quitting attempts with (OR=1.30; 95% CI: 1.00–1.68) or without (OR=3.54; 95% CI: 2.56–4.89) NSCS enrollment at the time of relapse, was significantly associated with the increased incidence of relapsing back to tobacco use. The incidence of participants who relapsed back into tobacco use was much higher among those who considered ‘reducing the amounts of tobacco used’ (OR=8.69; 95% CI: 6.10–12.38) and ‘having experience with quitting successfully for some periods of time’ (OR=2.14; 95% CI: 1.62–2.81) rather than participants who considered ‘increasing self-efficacy for successful tobacco cessation’ as the most helpful part of the NSCS.

**Table 3:** Factors associated with tobacco use at 12-month follow-up among the adult smokers registered in the NSCS in 2018 who succeeded in tobacco abstinence at baseline, results from a prospective follow-up, 2018–2020 (N=2129)

*Variables*	*Participants in tobacco abstinence at baseline^a^ (N=2129) n (%)*	*Participants in tobacco use at follow-up at 12 months^b^ (N=814) n (%)*	*OR (95% CI)*	*AOR (95% CI)*
**Gender**				
Male ®	1899 (89.2)	736 (38.8)	1	1
Female	230 (10.8)	78 (33.9)	0.81 (0.61–1.08)	0.82 (0.61–1.09)
**Age** (years)				
20–29 ®	202 (9.5)	70 (34.7)	1	1
30–39	311 (14.6)	115 (37.0)	1.11 (0.76–1.60)	1.10 (0.76–1.60)
40–49	486 (22.8)	195 (40.1)	1.26 (0.90–1.78)	1.26 (0.89–1.77)
≥50	1130 (53.1)	434 (38.4)	1.18 (0.86–1.61)	1.17 (0.86–1.60)
**Residence**				
Metropolitan ®	901 (42.3)	341 (37.8)	1	1
City and county	1228 (57.7)	473 (38.5)	1.03 (0.86–1.23)	1.03 (0.86–1.23)
**Most satisfied services in NSCS ever enrolled**				
Information on how to quit ®	201 (9.5)	74 (36.8)	1	1
Professional counseling/support	553 (26.2)	149 (26.9)	0.63 (0.45–0.89)	0.64 (0.45–0.90)
Medications/NRTs	1064 (50.4)	484 (45.5)	1.43 (1.05–1.96)	1.45 (1.06–1.98)
Gift/incentives/tool kits	250 (11.8)	84 (33.6)	0.87 (0.59–1.28)	0.90 (0.61–1.33)
Other	42 (2.0)	10 (23.8)	0.54 (0.25–1.15)	0.55 (0.25–1.17)
**Length of the longest tobacco abstinence ever kept during the NSCS program and 12-month follow-up** (months)				
<1 ®	121 (5.7)	98 (81.0)	1	1
1–3	189 (8.9)	142 (75.1)	0.71 (0.41–1.24)	0.71 (0.41–1.25)
3–6	285 (13.4)	200 (70.2)	0.55 (0.33–0.93)	0.55 (0.33–0.93)
>6	1534 (72.1)	374 (24.4)	0.08 (0.05–0.12)	0.08 (0.05–0.12)
**Experience of quit attempt during the 12-month follow-up**				
Never even though relapsed ®	531 (24.9)	267 (50.3)	1	1
Ever without NSCS enrollment	306 (14.4)	240 (78.4)	3.60 (2.61–4.96)	3.54 (2.56–4.89)
Ever with NSCS enrollment	423 (19.9)	237 (56.0)	1.26 (0.98–1.63)	1.30 (1.00–1.68)
Never as in prolonged abstinence	869 (40.8)	70 (8.1)		
**The most helpful contents of the NSCS ever enrolled for the current tobacco cessation**				
Increasing self-efficacy for successful tobacco cessation ®	535 (25.3)	145 (27.1)	1	1
Getting information on ways to quit tobacco	290 (13.7)	91 (31.4)	1.23 (0.90-1.68)	1.23 (0.90–1.69)
Reducing amounts of tobacco used	239 (11.3)	182 (76.2)	8.59 (6.03–12.23)	8.69 (6.10–12.38)
Having experience in successfully quitting for certain periods	400 (18.9)	177 (44.3)	2.14 (1.62–2.81)	2.14 (1.62–2.81)
Encouraging the possibility of repetitive quit attempt	606 (28.7)	196 (32.3)	1.29 (1.00–1.66)	1.30 (1.01–1.68)
Other	45 (2.1)	12 (26.7)	0.98 (0.49–1.95)	0.98 (0.49–1.95)
**Type of tobacco product used before quit attempt through NSCS**				
Heated tobacco ®	84 (3.9)	24 (28.6)	1	1
E-cigarettes	33 (1.6)	15 (45.5)	2.08 (0.91–4.79)	2.16 (0.94–4.98)
Conventional cigarettes	2008 (94.3)	774 (38.5)	1.57 (0.97–2.54)	1.57 (0.97–2.56)
Other tobacco product	4 (0.2)	1 (25.0)	0.83 (0.08–8.42)	0.83 (0.08–8.36)

a Participants who keep tobacco abstinence during and upon the completion of the NSCS program, which is the baseline when they were enrolled.

b Participants in tobacco use for the last seven days at follow-up at 12 months upon the completion of the last NSCS program enrolled. AOR: adjusted odds ratio; adjusted for age and gender. ® Reference categories. NRT: nicotine replacement therapy. NSCS: national smoking cessation services.

Table 4 presents the factors associated with tobacco cessation at 12-month follow-up among the participants who failed in tobacco abstinence at baseline. A total of 15.1% (n=460) of participants who were non-abstinent (i.e. had failed to quit) at baseline had succeeded in ceasing to use tobacco at the follow-up at 12 months. Being female, those with longer past experiences with tobacco abstinence (OR=6.67; 95% CI: 4.89–9.08 for six months or more) and with additional quitting attempts with NSCS enrollment during the 12-month follow-up showed a positive association with the 7-day point prevalence of tobacco cessation at the follow-up at 12 months. Those participants who answered that the most helpful part of the NSCS was ‘encouraging the possibility of repeated quitting attempts’ (OR=1.78; 95% CI: 1.10–2.86) had an increased 7-day point prevalence of tobacco cessation compared to those who answered that the most helpful part of the NSCS was the ‘increased self-efficacy for successful tobacco cessation’.

**Table 4:** Factors associated with tobacco cessation at 12-month follow-up among the adult smokers registered in the NSCS in 2018 who failed in tobacco abstinence at baseline, results from a prospective follow-up, 2018–2020 (N=3038)

	*Participants in non-abstinence at baseline^a^ (N=3038) n (%)*	*Participants in tobacco cessation at follow-up at 12 months^b^ (N=460) n (%)*	*OR (95% CI)*	*AOR (95% CI)*
**Gender**				
Male ®	2653 (87.3)	385 (14.5)	1	1
Female	385 (12.7)	75 (19.5)	1.43 (1.08–1.88)	1.42 (1.08–1.87)
**Age** (years)				
20–29 ®	431 (14.2)	70 (16.2)	1	1
30–39	499 (16.4)	72 (14.4)	0.87 (0.61–1.24)	0.88 (0.62–1.26)
40–49	702 (23.1)	99 (14.1)	0.85 (0.61–1.18)	0.86 (0.62–1.20)
≥50	1406 (46.3)	219 (15.6)	0.95 (0.71–1.28)	0.97 (0.72–1.30)
**Residence**				
Metropolitan ®	1314 (43.3)	209 (15.9)	1	1
City and county	1724 (56.7)	251 (14.6)	0.90 (0.74–1.10)	0.90 (0.74–1.10)
**Most satisfied services in NSCS ever enrolled**				
Information on how to quit ®	327 (11.1)	43 (13.1)	1	1
Professional counseling/support	817 (27.6)	116 (14.2)	1.09 (0.75–1.59)	1.10 (0.75–1.60)
Medications/NRTs	1347 (45.5)	209 (15.5)	1.21 (0.85–1.73)	1.25 (0.88–1.78)
Gift/incentives/tool kits	366 (12.4)	68 (18.6)	1.51 (1.00–2.28)	1.54 (1.01–2.34)
Other	102 (3.4)	12 (11.8)	0.88 (0.45–1.74)	0.89 (0.45–1.76)
**Length of the longest tobacco abstinence ever kept during the NSCS program and 12-month follow-up** (months)				
<1 ®	1020 (33.6)	58 (5.7)	1	1
1–3	688 (22.6)	88 (12.8)	2.43 (1.72–3.44)	2.46 (1.74–3.49)
3–6	498 (16.4)	83 (16.7)	3.32 (2.33–4.73)	3.44 (2.41–4.92)
>6	832 (27.4)	231 (27.8)	6.38 (4.70–8.65)	6.67 (4.89–9.08)
**Experience of quit attempt during the 12-month follow-up**				
Never even though relapsed	1351 (44.5)	0 (0.0)		
Ever without NSCS enrollment ®	1054 (34.7)	264 (25.0)	1	1
Ever with NSCS enrollment	633 (20.8)	196 (31.0)	1.34 (1.08–1.67)	1.41 (1.12–1.76)
Never as in prolonged abstinence	-	-	-	-
**The most helpful contents of the NSCS ever enrolled for the current tobacco cessation**				
Increasing self-efficacy for successful tobacco cessation ®	316 (11.8)	27 (8.5)	1	1
Getting information on ways to quit tobacco	503 (18.7)	14 (2.8)	0.31 (0.16–0.59)	0.31 (0.16–0.59)
Reducing amounts of tobacco used	686 (25.5)	14 (2.0)	0.22 (0.12–0.43)	0.22 (0.12–0.43)
Having experience in successfully quitting for certain periods	541 (20.1)	29 (5.4)	0.61 (0.35–1.04)	0.69 (0.40–1.20)
Encouraging the possibility of repetitive quit attempt	511 (19.0)	68 (13.3)	1.64 (1.03–2.63)	1.78 (1.10–2.86)
Other	131 (4.9)	1 (0.8)	0.08 (0.01–0.61)	0.08 (0.01–0.60)
**Type of tobacco product used before participating in NSCS**				
Heated tobacco ®	155 (5.1)	32 (20.6)	1	1
E-cigarettes	53 (1.7)	12 (22.6)	1.13 (0.53–2.39)	1.06 (0.50–2.26)
Conventional cigarettes	2828 (93.1)	416 (14.7)	0.66 (0.44–0.99)	0.63 (0.42–0.95)
Other tobacco product	2 (0.1)	0 (0.0)	<0.001	<0.001

a Participants who failed to keep the tobacco abstinence during and upon the completion of the NSCS program, which is the baseline when they were enrolled.

b Participants in tobacco abstinence for the last seven days at follow-up at 12 months upon the completion of the last NSCS program enrolled. AOR: adjusted odds ratio; adjusted for age and gender. ® Reference categories. NRT: nicotine replacement therapy. NSCS: national smoking cessation services.

## DISCUSSION

This study is the first to assess the overall effect of the NSCS on cessation outcome after the program and at 12-month follow-up among the Korean population based on actual data of NSCS-registered participants at the national level. From the results of the present study, we noted a relatively higher incidence of long-term tobacco cessation among NSCS participants, particularly those who succeeded in abstinence upon completion of the NSCS program.

Although the tobacco abstinence rates among NSCS participants were self-reported, with the possibility of overestimation when compared with biologically verified studies, the long-term abstinence rate was much higher than those previously reported in other countries, such as the 9.3% CO-validated long-term abstinence rate at 52 weeks (14.7% with the inclusion of self-reported cases without CO validation) in England and the 9.1% long-term abstinence rate at the 12-month follow-up among Quitline telephone service participants in the United States^3,9^. These results indicate that NSCS enrollment is effective in not only improving quitting outcomes upon completion of the NSCS program but also prolonging tobacco cessation for up to one year after participation in the NSCS.

Furthermore, of the smoking behaviors examined in this study, the increased number of quitting attempts and the experience of longer durations of abstinence were positively associated with long-term abstinence at the follow-up at 12 months. Longer past experiences with tobacco abstinence and multiple quitting attempts with appropriate help from cessation services during the follow-up period were identified as significant predictors for increasing the 7-day point prevalence of tobacco cessation at the follow-up at 12 months. The findings in this study reflect those demonstrated in other studies that many smokers attempt to quit and relapse a number of times before they achieve sustained abstinence^10,11^. Also, previous studies indicated that the duration of abstinence is an important predictor of continued long-term abstinence^12-14^. Tombor et al.^10^ reported evidence from a population survey in England that increased length of abstinence at baseline, which was the only significant predictor of long-term abstinence, showed a significant OR of 1.41 (95% CI: 1.13–1.76) for long-term abstinence (at the 6-month follow-up) compared to smokers who relapsed.

This study also found that additional attempts to quit, regardless of NSCS enrollment after relapse, could result in negative outcomes in lasting tobacco cessation. This might introduce the necessity of offering assisted and tailored cessation programs to encourage readiness to quit again and establish proper schedules for quitting. Further studies to verify these possibilities should be undertaken.

The most satisfactory contents of the NSCS were also suggested as factors associated with the 7-day point prevalence of tobacco cessation at the follow-up at 12 months. Citing professional counseling/support and prescription medications/NRTs as the most satisfactory contents of the NSCS were highlighted as predictors of higher 7-day point prevalence of tobacco cessation at the follow-up at 12 months. Our results support the conclusions of previous studies. A study conducted in the United States also showed that incentives were helpful to assist smokers in maintaining their abstinence at the 4- to 6-month follow-up. Similarly, participants in the incentive group had significantly higher rates of smoking cessation than did participants in the information-only group 9 or 12 months after enrollment (14.7% vs 5.0%)^15^. Among smokers who enrolled in ‘Quit and Win’ incentive-based stop-smoking contests, the self-reported quitting rates (based on the 7-day point prevalence) ranged from 22–49%, with an average of 31% at the 4- to 6-month follow-up^16^. Therefore, offering incentives for tobacco cessation not only recruits smokers and encourages them to make serious attempts to quit but also helps them to quit and maintain their abstinence, which should be considered for future NSCS offerings to prolong tobacco cessation.

However, 38.2% of smokers who had successfully quit at baseline had relapsed at the follow-up at 12 months. These findings were similar to those of a previous study reporting a relapse rate of 33% after a 5-year follow-up^17^. In the other studies conducted in England, the United States, and Shanghai, China, the relapse rates within the first year of smoking cessation ranged from 70–90%^5,18,19^. When the most satisfactory services noted among the tobacco abstinence group at baseline were NRTs or prescribed medications, participants were more likely to relapse. These results could be explained by the fact that baseline tobacco abstinence, while highly relying on cessation drugs, might not continue once the uptake of drug use is stopped, as suggested in previous studies^20^. Relapse at the 12-month follow-up was more likely among those who succeeded in reducing the amount of tobacco they used and those who quit successfully for certain periods compared to participants with increased self-efficacy for successful tobacco cessation. Participants may feel that they have taken sufficient positive steps and may have less desire to quit completely or for longer periods of time than smokers with helpful experience in increasing self-efficacy for successful tobacco cessation. These results suggest the importance of increasing participant self-efficacy to initiate successful long-term cessation outcomes and to make successful quitting outcomes of NSCS enrollment.

Although some previous studies have suggested that gradual reduction of or cutting down tobacco use before quitting are helpful cessation methods for providing motivation to quit among smokers who have experienced difficulties in quitting abruptly, most population-based studies have suggested that abrupt quitting is more likely to lead to lasting abstinence than cutting down first^21-23^. In such a situation, if the smokers are satisfied with their attempts and/or progress in quitting tobacco, even if they have not yet become non-smokers or completely quit smoking, it can negatively affect their long-term abstinence, increasing the incidence of relapse. Therefore, unintended consequences can occur when promoting interventions, as many people believe that reducing the number of cigarettes smoked or successfully quitting for certain periods of time are helpful, satisfactory, and even signs of success. Therefore, it is necessary to let smokers know that reducing the number of cigarettes smoked or having successfully quit smoking for certain periods of time should not be recognized as sufficient outcomes of attempting to quit but should instead be seen as steps in the process towards complete and permanent tobacco use cessation. Moreover, the potential effects of smokers’ recognition and understanding in the context of the process of tobacco use cessation and the assistance offered by cessation services should be explored with caution and with the aim of encouraging prolonged cessation or future attempts to quit.

Finally, 15.1% of participants in the non-abstinence at baseline group had quit using tobacco at the follow-up at 12 months. Among participants who used the NSCS but failed to maintain their tobacco abstinence at baseline, those with helpful experiences in encouraging the possibility of repeated attempts to quit from the NSCS could maintain long-term cessation. As is well-known and suggested in a previous study, a past quitting attempt and some amount of success in quitting are necessary for most smokers before they are eventually able to maintain their prolonged abstinence from smoking^24,25^. The current study results also indicated that positive experiences from the NSCS, such as encouraging the possibility of repeated attempts to quit, can promote attempting to quit and achieving success at a later date, even among smokers who fail to maintain their smoking cessation upon completion of the NSCS program. Moreover, smokers need to understand what should be pursued during the process and after the completion of the NSCS program to better promote potential quit attempts and prolong smoking cessation over time.

### Limitations

Although the current findings suggested the effects of NSCS enrollment on both long-term tobacco cessation and the change in tobacco cessation status from baseline to the follow-up at 12 months, with a prospective follow-up design in a real-world setting, several limitations exist. First, the available information and the contents collected varied based on the type of cessation services included in the NSCS. Although only the variables that were considered for all NSCS were included in the analysis, efforts were made to harmonize and standardize the variability in the information during data cleansing and processing, which could have affected the results of the current study. Second, successful tobacco cessation was defined according to the definitions defined by the type of cessation services included in the NSCS, as the length of the service delivery and the criteria for determining successful quitting differed by NSCS program. Third, this study may have limited generalizability to other countries due to differences in available cessation services and tobacco use behaviors in Korea. Fourth, long-term tobacco cessation at the 12-month follow-up was determined by self-report without CO measurements or any other objective measures, which could be better for verification of abstinence. It is possible that participants could have displayed recall or social desirability bias. Meanwhile, self-reported smoking status has been well matched to biologically verified status and has been demonstrated to have high validity in some previous studies^26,27^. Finally, this study could be affected by residual confounding due to a lack of data availability by adjusting only available confounders. Comprehensive analysis in the future is needed to replicate these findings in larger samples and include various confounders to minimize residual bias in determining the transitions of abstinence and relapse.

## CONCLUSIONS

Findings from this study identified the positive effect of NSCS enrollment on long-term tobacco cessation at the follow-up at 12 months, regardless of the quitting outcomes upon the completion of the NSCS program. In agreement with previous evidence, it was shown that multiple quitting attempts, longer experience with tobacco abstinence, and additional enrollment in NSCS might improve the lasting success of abstinence at the follow-up at 12 months. Levels of satisfaction with the contents and services offered during the NSCS program, such as professional counseling/support, prescription medications, etc., could affect the transition of tobacco cessation status from baseline to the 12-month follow-up differently. These results might be considered to improve the contents and protocols of the NSCS to increase their quality and provide better outcomes. Further studies are necessary to develop tailored behavioral interventions that can be effective in helping people achieve long-term tobacco cessation, including relapse prevention, in population-based cessation service offerings.

## Data Availability

The data underlying this article cannot be shared publicly due to privacy of individuals that participated in the study.
