# Supplementary material for: National Smoking Cessation Services (NSCS) enrollment and their effect on long-term tobacco cessation in Korea: Results from a 1-year prospective follow-up of NSCS participants
Source: Tob Induc Dis. 2024 Feb 8;22:10.18332/tid/178499. doi: 10.18332/tid/178499 (PMC10851190; doi:10.18332/tid/178499)
Supplement: Supplementary file 1 [file TID-22-33-s1.pdf]

Supplementary Material

Supplementary Table 1. The number of National Smoking Cessation Services (NSCS) users in 2018 and the number of participant enrolled in the current study with the stratified proportionated random sampling by the type of NSCS

	Total participants (n)	%	Target (n)	Final # enrolled (n)
Public health center-based smoking cessation Clinics (PHC)	515,126	55.2	2,854	**2,785**
Quitline (QL)	3,019	0.3	17	**23**
Mobile smoking cessation clinics (MC)	21,232	2.3	117	**176**
Individual tailored intensive smoking cessation services in hospital (ITIS)	4,074	0.4	23	**34**
General smoking cessation services (GECS)	2,039	0.2	11	**8**
Smoking cessation treatment services in hospitals/clinics (TSH)	387,108	41.5	2,145	**2,141**
Total	932,598	100	5,167	**5,167**

© 2024 Park J. et al.
